# Ecological correlation between short term exposure to particulate matter and hospitalization for mental disorders in Shijiazhuang, China

**DOI:** 10.1038/s41598-023-37279-7

**Published:** 2023-07-14

**Authors:** Lan Wang, Xian Gao, Ran Wang, Mei Song, Xiaoli Liu, Xueyi Wang, Cuixia An

**Affiliations:** 1 Mental Health Center, The First Hospital of Hebei Medical University, No. 89 Donggang Road, Shijiazhuang, 050031 China; 2 Hebei Clinical Research Center for Mental Disorders and Institute of Mental Health, Shijiazhuang, China; 3 Hebei technical Innovation Center for Mental Health assessment and Intervention, Shijiazhuang, China; 4 Department of Gastrointestinal Surgery, The First Hospital of Hebei Medical University, Shijiazhuang, China; 5 The third Hospital of Shijiazhuang, Shijiazhuang, China

**Keywords:** Psychosis, Psychology and behaviour

## Abstract

The associations between particulate matter (PM) and overall and specific mental disorders (MDs) are investigated using data from two general hospitals in Shijiazhuang, China, from January 2014 to December 2019. A longitudinal time series study, as one type of ecological study, is conducted using a generalized additive model to examine the relationship between short-term exposure to PM2.5, PM10, and daily hospital admissions for MDs, and further stratification by subtypes, age, and gender. A total of 10,709 cases of hospital admissions for MDs have been identified. The significant short-time effects of PM2.5 on overall MDs at lag01 and PM10 at lag05 are observed, respectively. For specific mental disorders, there are substantial associations of PM pollution with mood disorders and organic mental disorders. PM2.5 has the greatest cumulative effect on daily admissions of mood disorders and organic mental disorders in lag01, and PM 10 has the greatest cumulative effect in lag05. Moreover, the effect modification by sex or age is statistically significant, with males and the elderly (≥ 45 years) having a stronger effect. Short-term exposure to PM2.5 and PM10can be associated with an increased risk of daily hospital admissions for MDs.

## Introduction

Mental disorders are a class of syndromes characterized by clinically significant dysfunctions in cognition, emotion regulation, or behavior. The etiology of mental disorders is unknown, and its pathogenesis is closely related to genetic-environmental factors. Environmental pollution has been identified as a risk factor for mental health^1^. It had been found that air pollution can exacerbate symptoms of Alzheimer’s disease, schizophrenia, psychiatric emergencies, or increase emergency hospital admissions in Spain^2^, Japan^3^, Italy^4^, etc. A total of 20,000 residents in 25 regions in China were surveyed from 2010 to 2014^5^, and the result revealed that increases in air pollution and temperature changes were associated with an increase in mental health problems. In a review, Omar also described increasing evidence that proinflammatory mediators and reactive oxygen species can affect the health of the central nervous system and brain after exposure to various air pollutants, leading to the occurrence of various MDs^6^.Particulate matter (PM) is an important component of air pollution, and its link to mental health is receivingincreasing attention. PM_10_, PM_2.5_, PM_1_, black carbon, smoking and cooking fumes, and so on are all examples of PM. PM_2.5_ has a large surface area and a small diameter than PM_10_. It can suspend in the air for a long time and travel long distances. In the air, various harmful substances, such as heavy metals and polycyclic aromatic hydrocarbons, are easily absorbed. Although both PM_2.5_ and PM_10_ are inhalable particles, some of PM_10_ can be excreted through sputum and blocked by the villi inside the nasal cavity; PM_2.5_, on the other hand, can partially enter the gas exchange area of the human lungs, pass through the respiratory barrier, enter the circulatory system, and spread throughout the body, making PM_2.5_ more harmful to the human body than PM_10_. Shijiazhuang, one of the most polluted cities in northern China, under went a study from 2014 to 2016 that focused on the effect of PM_2.5_ and PM_10_ on the daily admission rates for mental and behavioral disorders^6^. It demonstrated that various air pollutants, particularly PM and monoxide nitrogen, were related to poor mental health. However, the study was conducted over a short period of time, and the effects of PM on the subtypes of MDs were unclear. Therefore, a longer study of the relationship between air pollution and MDs in Shijiazhuang will be required, as will further research into the effect of PM on specific MDs. We collected air quality data and hospital admissions for MDs from 2014 to 2019 to investigate the impact of air pollution on hospital admissions for general and specific MDs using time series regression analysis.

## Methods

### Data collection

Shijiazhuang City is the capital of Hebei Province, which is located in the southwest of Hebei Province, China, at latitude 114°26′E and longitude 38°03′N. As of the end of 2019, the permanent population was 10.39 million in Shijiazhuang. It has to a typical temperate monsoon climate with distinct seasonal fluctuations. This study was performed in accordance with the tenets of the Declaration of Helsinki and was approved by the ethics committee of the First Hospital of Hebei Medical University.

The data were retrieved from the First Hospital of Hebei Medical University and the Third Hospital of Shijiazhuang, both of which were general hospitals in Shijiazhuang. During the study period, the patients admitted to the hospital due to mental disorders were pulled from the hospital information system every day (January 1, 2014, to December 31, 2019). Mental disorders were classified using the International Classification of Diseases-10th Edition (ICD-10).

This study was an ecological study and applied the average method to evaluate the level of air pollutants. The average method refers to calculating the average value of certain air pollutants at air detection stations in a certain area as the exposure value for all research objects in that area.The daily air pollution data forgaseous pollutants such as PM_2.5_ (μg/m^3^) and PM_10_ (μg/m^3^) were obtained from the Ministry of Environmental Protection of China’s website (https://www.aqistudy.cn/). The daily average concentration of air pollutants was recordedat seven fixed stations in the traditional urban area of Shijiazhuang City. These stations are required to be located far away from major roadways, industrial sources, buildings, or domesticcoal, oil, or trashburning emission sources, with a good reflection of the level of air pollution in the city. Daily weather data, including daily average temperature (°C) and relative humidity (%), came from the China Meteorological Data Sharing Service System (http://data.cma.cn/).

### Statistical analyses

We applied a time-series approach to analyze the data, which has the advantage of automatically controlling for time-invariant confounders at the population level. An over-dispersed generalized additive model (GAM) was applied to analyze the association between PM (PM_2.5_ and PM_10_) and daily hospital admissions for mental and behavioral disorders. Several covariates were introduced to control for potential confounding effects: (1) a natural cubic regression smooth function of calendar time with sevendegrees of freedom (df) per year (2) natural smooth functions of the mean temperature (6, df) and relative humidity (3, df) to account for the nonlinear confounding effects of weather conditions; (3) indicator variables for “day of the week”. The main model is described as follow: logE(Yt) = βZt + ns (time, df) + ns(temperature, 6) + ns(humidity, 3) + DOW + intercept, where E(Yt) represents the expected number of hospital admissions for mental and behavioral disorders at day t; β represents the log-related rate of mental and behavioral disorders admission rate associated with a unit increase of PM pollutants; Zt represents the pollutant concentrations at day t; DOW is a dummy variable for the day of the week; ns indicates natural cubic regression smooth function. After the basic model was established, we further introduced both single-day lags from 0 to 7and moving average exposure of multiple days, including lag0–1, 0–2, 0–3, 0–4, 0–5, 0–6, 0–7. The exposure-response relationship curves between PM_2.5_, PM_10_, and hospital admissions for mental and behavioral disorders were plotted by including a natural spline function with 3 df in the above model. Two sensitivity analyses were performed to ensure the stability of our model. First, we selected alternative df with 4–10 per year for the smoothness of time trends. Second, we created two-pollutant models to examine the robustness of the effect estimates after adjusting for co-pollutants.

Furthermore, we conducted stratification analyses to explore the potential effect of modification by age (<45, ≥45)^7^, and sex. We further evaluated the statistical significance for the differences in estimates across strata by calculating 95% confidence intervals (CI) as ($\widehat{\mathrm{Q}}1-\widehat{\mathrm{Q}}2)\pm 1.96 \sqrt {S\hat{E}_{1}^{2} + S\hat{E}_{2}^{2} }$,where $\widehat{\mathrm{Q}}1$ and $\widehat{\mathrm{Q}}2$ are the estimates for two categories, $\mathrm{and}$ $S\hat{E}_{1}^{2}$ and $S\hat{E}_{2}^{2}$ are their standard errors.

The statistical tests were two-sided, and effects of *P*<0.05 were considered statistically significant. All statistical models were run in R software (version 4.1.1) using the MGCV package. Excess risk (ER)=(OR–1)×100%, which represents the percentage of change in daily hospital admissions for mental and behavioral disorders per 10 μg/m^3^ increase of PM (PM_2.5_ and PM_10_).

### Ethics approval and consent to participate

This study was performed in accordance with the tenets of the Declaration of Helsinki and was approved by the ethics committee of the First Hospital of Hebei Medical University. All participants provided written informed consent before admission.

## Results

### Data description

**Figure 1:**
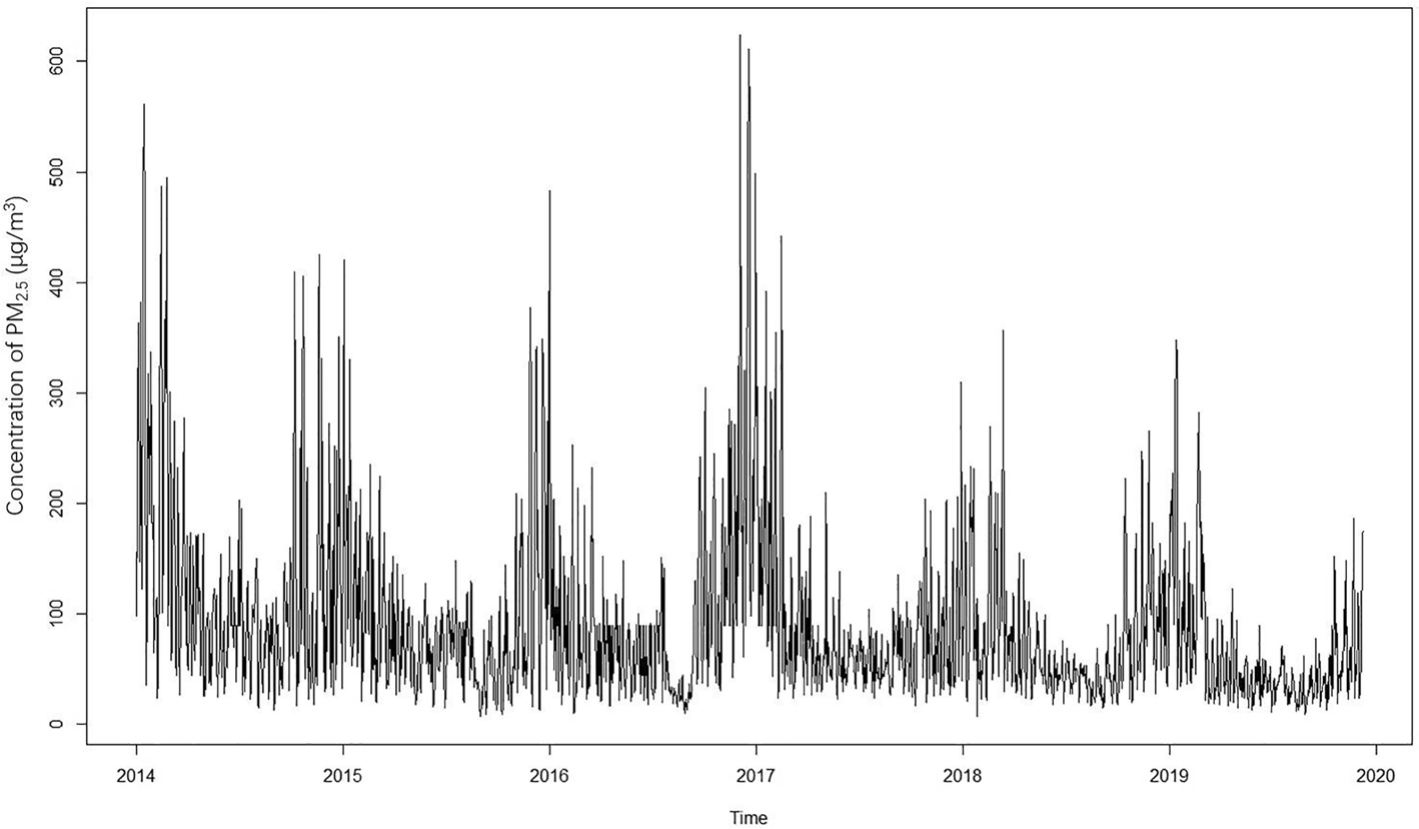
Distribution of PM_2.5_ from 2014 to 2019.

From January 1st, 2014 to December 31st, 2019, a total of 10,709 patients met the ICD-10 diagnostic criteria for mental disorders in the two hospitals, with 32.99% being males and 54.51% being 45 years or younger. The daily average PM_2.5_ and PM_10_ concentrations were 89.25 and 157.16μg/m^3^, respectively, the number of days abovethe average daily PM_2.5_ (≥75μg/m^3^) concentrations in Chinaper year was 240, 177, 164, 146, 140, 89 (the 6-year average was 159) from 2014 to 2019, while 234, 146, 141, 148, 125, 93 (the 6-year average was 148) for PM_10_ (≥150μg/m^3^), respectively. The daily average temperature was 14.98 °C, with a humidity of 55.15%. As shown in Table 1, the distributions of data on daily admissions for mental disorders and subtypes are listed. The distribution of PM_2.5_ from 2014 to 2019 is shown in Fig. 1. And the distribution of PM_10_, SO_2_, CO, NO_2_, O_3_ is displayed in Supplementary Figs. 1 to 5, respectively.

**Table 1:** The summary of descriptive statistics during the study period (January 1st, 2014 to December 31st, 2019).

	Mean	SD	Min	P25	Median	P75	Max
Sex distribution of daily admissions (n)							
Male	2.21	6.19	0	0	0	2	75
Female	4.49	7.69	0	1	3	5	89
Age distribution of daily admissions (year)							
< 45	3.65	7.57	0	1	2	4	101
≥ 45	3.05	6.17	0	1	1	3	76
Air pollutant concentration (μg/m^3^)							
PM_2.5_	89.25	77.93	6.53	38.89	65.72	107.78	623.55
PM_10_	157.16	110.54	9.86	84.31	130.79	190.66	864.14
Meteorological measures							
Temperature (℃)	14.98	20.28	− 9.40	4.83	16.40	24.80	35.50
Humidity (%)	55.15	20.28	12.00	39.00	55.00	71.00	100.00
No. of daily admissions for mental disorders (n)	6.69	13.98	1	2	3	6	163
No. of daily admissions for schizophrenia^a^ (n)	1.49	2.54	0	0	1	2	30
No. of daily admissions for mood disorders (n)	3.38	6.46	0	1	2	4	80
No. of daily admissions for anxiety disorders^b^ (n)	0.39	1.30	0	0	0	0	20
No. of daily admissions for organic mental disorders (n)	0.66	2.03	0	0	0	0	29
No. of daily admissions for other mental disorders (n)	1.35	4.21	0	0	0	0	64

^a^also include other primary psychotic disorders. Mood disorders include manic episodes, bipolar disorder, depressive episodes, etc. ^b^also include and fear-related disorders, obsessive-compulsive disorders, somatoform disorder, dissociation (conversion) disorder. Organic mental disorders include Alzheimer’s disease, vascular dementia, delirium, etc*.* Other mental disorders include mental and behavioral disorders caused by alcohol, stress-related disorders, personality disorders, sleep disorders, neurodevelopmental disorders, etc*.*

#### Single-pollutant model analysis

**Figure 2:**
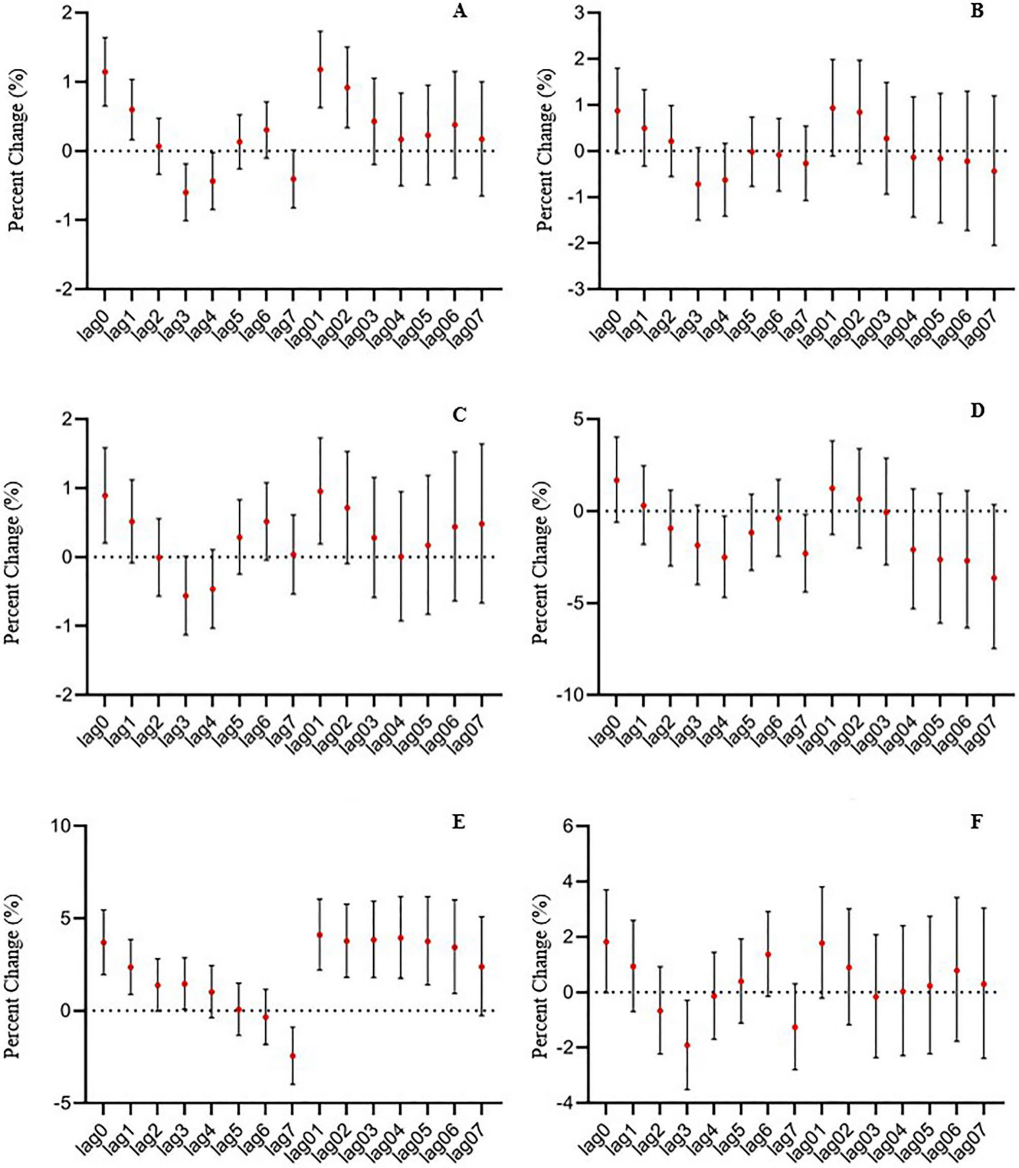
Association between PM_2.5_ and daily hospital admission for mental disorders, Shijiazhuang, China, 2014–2019. Results express as percentage change (95% CI) in daily hospital admission per 10 μg/m^3^ increase in PM_2.5_ concentration. (**A**) all mental disorders; (**B**) schizophrenia and other primary psychotic disorders; (**C**) mood disorders (including manic episodes, bipolar disorder, depressive episodes, etc.); (**D**) anxiety disorders and fear-related disorders, obsessive-compulsive disorders, somatoform disorder, dissociation (conversion) disorder; (**E**) organic mental disorder (including Alzheimer’s disease, vascular dementia, delirium, etc.), (**F**) other mental disorders (including mental and behavioral disorders caused by alcohol, stress-related disorders, personality disorders, sleep disorders, neurodevelopmental disorders, etc.).

Figure 2A depicted the lag–response relationships for the impact of PM_2.5_ on the daily hospital admissions for mental disorders. The lag effect of PM_2.5_ on daily admissions for all mental disorders was statistically significant at lag0 and lag1, and its cumulative effect was greatest at lag01. The excess risk (ER) value of admission for mental disorders was 1.18% for every 10g/m^3^ increase in PM_2.5_ concentration(95% CI 0.63–1.73%).

The results of the exposure-response relationship between specific mental disorders and PM_2.5_ were inconsistent in the subgroup of mental disorders, with statistically significant differences only in mood disorders and organic mental disorders (Fig. 2B–F). The lag effect of PM_2.5_ on daily hospital admission for mood disorders/organic mental disorders was statistically significant at lag0/lag0, lag1, and the greatest cumulative effect was at lag01, with the statistical significance for mood disorders at lag01 and organic mental disorders at lag01-lag06. The effect of PM_2.5_ on daily admissions of schizophrenia and primary psychotic disorders, anxiety and related disorders, and other mental disorders was similar to the trend of the aforementioned diseases, but there was no statistically significant difference.

**Figure 3:**
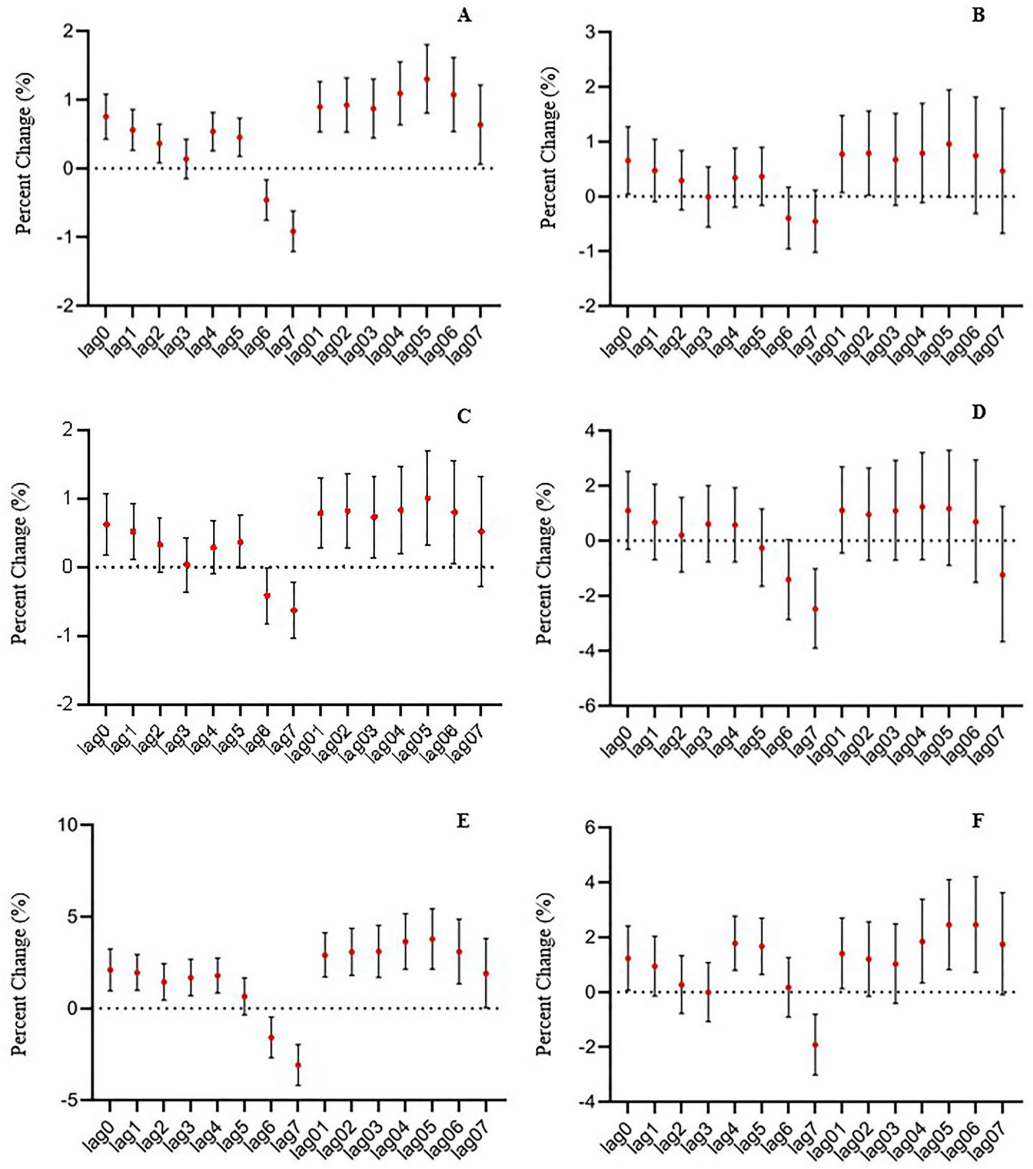
Association between PM_10_ and daily hospital admission for mental disorders, Shijiazhuang, China, 2014–2019. Results expressed as a percentage change (95% CI) in daily hospital admission per 10 μg/m^3^ increase in PM_10_ concentration. (**A**) all mental disorders; (**B**) schizophrenia and other primary psychotic disorders; (**C**) mood disorders (including manic episodes, bipolar disorder, depressive episodes, etc*.*); (**D**) anxiety disorders and fear-related disorders, obsessive-compulsive disorders, somatoform disorder, dissociation (conversion) disorder; (**E**) organic mental disorder (including Alzheimer’s disease, vascular dementia, delirium, etc*.*), (**F**) other mental disorders (including mental and behavioral disorders caused by alcohol, stress-related disorders, personality disorders, sleep disorders, neurodevelopmental disorders, etc.).

The lag effect of PM_10_ on all mental disorders was evident in Fig. 3A, with statistical differences at lag0, lag1, and lag2, and the peak of the cumulative effect of PM_10_ was at lag05. The ER value of admission for mental disorders was 1.01% for every 10μg/m^3^ increase in PM_10_ concentration (95%CI 0.32–1.71%).

The results of the exposure-response relationship among specific mental disorders and PM_10_ were analogous to PM_2.5_ in the subgroup of mental disorders(Fig. 3B–F). The lag effect of PM_10_ on the daily hospital admission for mood disorders/organic mental disorders was statistically significant at lag0/lag0 to lag4, and the greatest cumulative effect were both at lag05, with statistical significance at lag01-lag06. In addition, statistically significant lag effects of PM_10_ on the daily admission for schizophrenia and primary psychotic disorders and other mental disorders were found at lag0, butonly cumulative effect at lag01. However,there was no statistically significant difference between PM_10_ and anxiety and related disorders.

**Figure 4:**
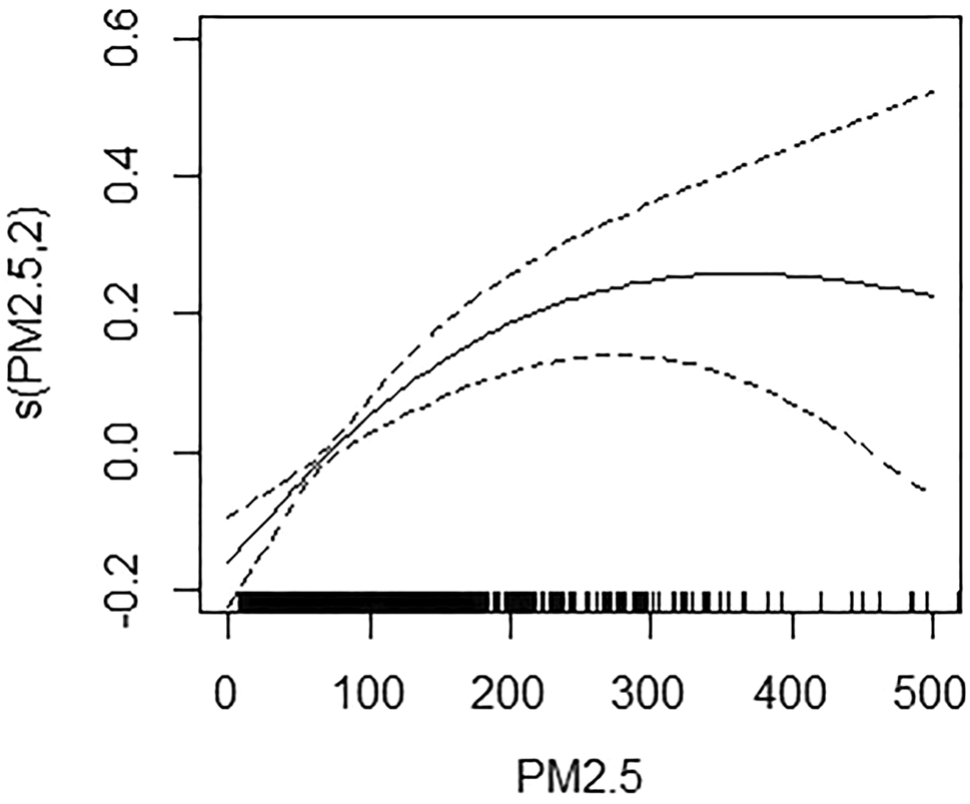
The exposure-response relationship curves for the lag01 day concentrations of PM_2.5_ with daily hospital admissions for mental disorders. The solid line represents mean estimates, and the dashed lines for 95% confidence intervals.

**Figure 5:**
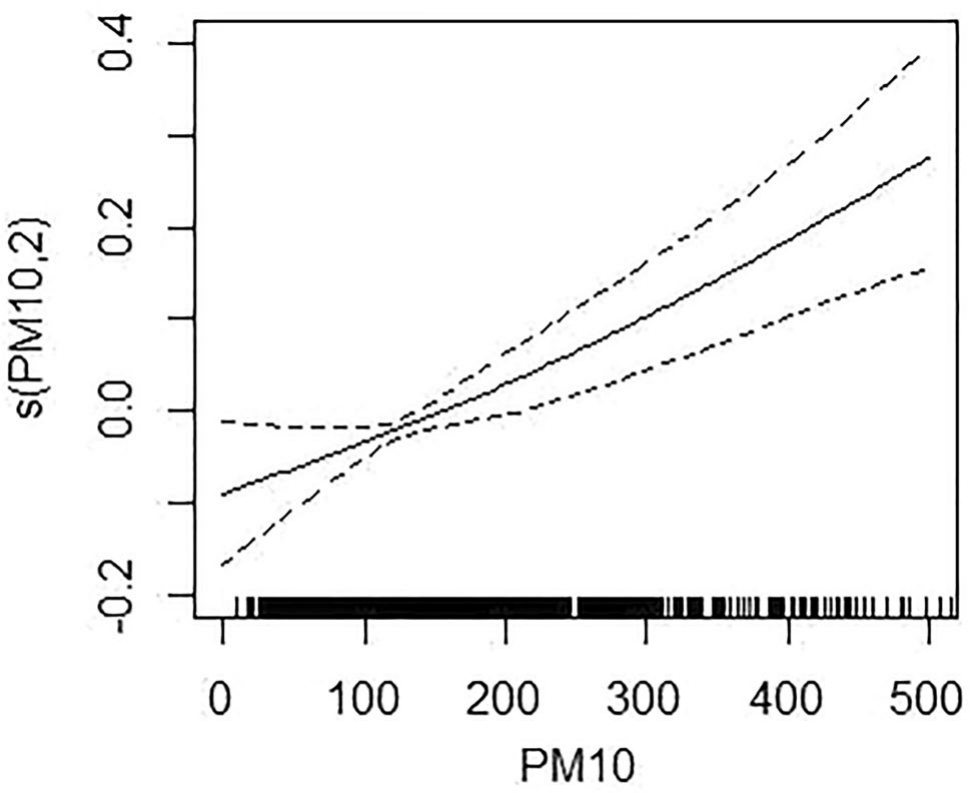
The exposure-response relationship curves for the lag05 day concentrations of PM_10_ with daily hospital admissions for mental disorders. The solid line represents mean estimates, and the dashed lines for 95% confidence intervals.

The daily hospitalization of PM and mental disorders was associated with the exposure-response relationship curve when accumulating lag 01 (PM_2.5_) and 05 (PM_10_), respectively, as shown in Figs. 4 and 5. Both the curves of PM_2.5_ and PM_10_ tended to alinear trend, indicating that there was no threshold for the association between PM_2.5_ or PM_10_ and daily hospitalization for mental disorders.The results of PM_2.5_ and PM_10_ sensitivity analyses confirmed that our models were stable at Table 2.

**Table 2:** Percent change (95% CI) of hospital admission for mental and behavioral disorders per 10 μg/m^3^ increase in pollutant concentrations at lag02 day using different degrees of freedom per year.

df	PM_2.5_	PM_10_
4	0.91 ( 0.35,1.46)	1.62 (1.17, 2.09)
5	0.93 (0.36,1.50)	1.43 (0.96, 1.09)
6	1.06 (0.50,1.64)	1.47 (0.99, 1.95)
7	1.03 (0.46,1.61)	1.47 (0.99, 1.97)
8	1.18 (0.59,1.77)	1.67 (1.17, 2.17)
9	1.39 (0.80,1.99)	1.98 (1.48, 2.49)
10	1.37 (0.78,1.96)	1.96 (1.45, 2.47)

### Effects by sex and age

PM_2.5_ and PM_10_ were significantly positively correlated with changes in the admission for mental disorders in both males and females, but more so in males. Table 3 shows a positive relationshipbetween PM and hospitalization for mental disorders in younger (less 45-year old) and older (45-year and more) people, which was more pronounced in the elderly.

**Table 3:** Percent change (95% CI) in hospital admission for mental disorders per 10 μg/m^3^ increase in concentrations of PM_2.5_ and PM_10_ stratified by gender and age in Shijiazhuang, China, 2014–2019.

Pollutant	Sex		Age	
	Male	Female	< 45	≥ 45
PM_2.5_	1.37 (0.24, 2.52)	0.90 (0.23, 1.57)	0.92 (0.14, 1.70)	1.21 (0.37, 2.06)
PM_10_	2.15 (1.24, 3.08)	1.23 (0.66, 1.80)	1.20 (0.56, 1.86)	1.83 (1.12, 2.54)

### Two-pollutant models analysis

In the two-pollutant model of PM_2.5_ and SO_2_, CO, NO_2_, and O_3_, there were significant positive correlations between the change of mental disorders admission at cumulative lag01, with statistically significant differences. The results of PM_10_ and SO_2_, CO, NO_2_, and O_3_ were similar to PM_2.5_ in the dual pollutant model at lag05, with statistically significant differences, as Table 4 showed.

**Table 4:** Percent change (95% CI) in hospital admission for mental disorders in two-pollutant models.

Two-pollutant models		lag	ER (95%CI)
PM_2.5_	SO_2_	01	1.57 (1.07, 2.08)
	CO	01	0.68 (0.02, 1.34)
	NO_2_	01	1.51 (1.03, 2.01)
	O_3_	01	1.14 (0.60, 1.67)
PM_10_	SO_2_	05	2.10 (1.56, 2.64)
	CO	05	4.05 (3.16, 4.93)
	NO_2_	05	1.50 (1.01, 1.99)
	O_3_	05	3.36 (2.64, 4.07)

SO_2_ sulfur dioxide, CO carbon monoxide, NO_2_ nitrogen dioxide, O_3_ ozone.

## Discussion

Similar to previous studies^7^, our study found that PM_2.5_ and PM_10_ were positively correlated with the daily hospital admission for mental disorders in Shijiazhuang from 2014 to 2019, but our findings suggested that the cumulative effect of PM_2.5_ was the most obvious at lag01, as the risk for mental disorders admissions increases (excess risk, ER) 1.18% (95% CI 0.63–1.73%) for every 10 μg/m^3^ increase in PM_2.5_ concentration; PM_10_ has the greatest cumulative effect at lag05, whose ER value for admission to the mental disorders was 1.01% (95% CI 0.32–1.71%) with a 10μg/m^3^ increase of PM_10_, which were significantly higher than the results of Song et al.^7^. They showed that for every unit increase in PM_2.5_ and PM_10_ concentrations (both lag02), the daily admissions for mental disorders increased by 0.48% (95% CI 0.18–0.79%) and 0.32% (95% CI 0.03–0.62%), respectively. Although their study was also conducted in Shijiazhuang, our study was longer, contained 6 years of data, and covered admissions for mental disorders in two comprehensive tertiary hospitals, with a bigger sample size. These may lead to discrepancies between the two studies. A study conducted in Shenzhen, China, also showed that as the concentration of air pollutants increase, so did the number of daily outpatient visits for mental disorders, such as PM_2.5_ at lag0 (ER=1.20%, 95% CI 0.28–2.13%), PM_10_ (ER = 0.99%, 95% CI 0.36–1.62%)^8^. The study of Chengdu from 2015 to 2016 displayed that at lag06 PM_2.5_ and PM_10_ increased by 10μg/m^3^, the daily hospitalization for mental disorders increased by 2.89% (95% CI 0.75–5.08%), 1.91% (95% CI 0.57–3.28%), respectively^9^. According to another survey conducted in Chengdu between 2013 and 2017, the exposure-response effects of PM pollution on hospital admissions for the overall and specific mental disorder were strong, at the cumulative lag03 day would be 3.25% (95% CI 2.34–4.16%) for PM_2.5_, and 6.38% (95% CI 4.79–7.97%) for PM_10_^10^. The results differed slightly from city to city, presumably due to climate differences. Thus, both PM_2.5_ and PM_10_ can increase the risk of daily hospital admissions for overall mental disorders, with PM_2.5_ having a more immediate effect than PM_10_.

Moreover, we analyzed the impact of PM on the daily admission in specific mental disorders. The results showed that the daily admission for mood disorders and organic mental disorders accumulated at lag01(PM_2.5_) and lag05(PM_10_) during short-time PM exposures. The effect of PM_2.5_ and PM_10_ had the strongest influences, which were the same as the overall mental disorder, but there was no statistical difference in the influence on schizophrenia and other primary psychotic disorders, anxiety disorders, and other mental disorders. A study in Madrid also showed that PM_2.5_ concentration at lag2 was related to Alzheimer's disease admission (RR=1.38, 95% CI 1.15–1.65)^2^; Similar results have been found in Zhejiang, China, where the risk of AD rised by 2%-5% per increase 10 μg/m^3^ in PM_2.5_, from 2013 to 2017^11^. A survey of 26 cities in China from 2013 to 2015 found that PM was positively correlated with hospital admissions for depression, PM_10_ was the strongest at lag0, PM_2.5_ was highest at lag0 and lag5, and the elderly (over 65 years) were more sensitive to PM, which was consistent with our findings^12^. Moreover, the high level of PM was related to the elevated suicide events among major depressive disorder patients, which was proved by research from 2004 to 2017 in Korean^13^. A Japanese study suggested that short-term PM_2.5_ exposure was associated with worsening symptoms in hospitalized schizophreniapatients (1193 cases)^3^. In addition, asurvey (11,373 cases) conducted in Hefei, China, from 2014 to 2016, suggested that short-term NO_2_ exposure may be related to the increase in hospitalizations for schizophrenia^14^. However, Carugno et al. found increasing PM_10_ levels shifts the manic episode towards the depressive pole of the bipolar disorder spectrum without increasing the risk of psychotic symptoms at admission^15^. A UK Biobank study (2006–2010) found that for per per 10 μg/m^3^ increase in PM_2.5_, the risk of major depression and bipolar disorder increased by 2.26 and 4.99 times, respectively^16^. Positive associations between PM_2.5_ or PM_10_ and anxiety admissions were found in a Chinese multicity case-crossover study^17^, and the highest RR of emergency room visit for anxiety disorder due to PM_2.5_(RR=1.709) and PM_10_(RR=2.618) in South Korea^18^. These results differed from ours and could be attributed to differences in sample size and air pollution levels. A meta-analysis showed that short-term PM_10_ build up was significantly associated with suicide in the first 2 days^19^. Furthermore, a Canadian study discovered that PM_2.5_ and PM_10_ levels were positively correlated with emergency department visits for alcohol and drug abuse. In our study, due to the modest number of suicides and substance use disorders, they were not evaluated as subgroups.

Although there was a positive correlation between particulate pollution and the daily admission for mental disorders in both males and females, over 45 and less than 45 years old, this effect was more pronounced in men and over 45 years of age. The results were consistent with past studies^7,10,20^. Other research had shown that female patients were more vulnerableto PM^9,17^.

The two-pollutant model revealedthat PM_2.5_ and PM_10_ were positively correlated with SO_2_, CO, NO_2_, O_3_, and the daily hospital admission for mental disorders, with PM_10_ and CO having the largest influence. Similar to aprevious study in South Korea, the two-pollutant model exhibiteda slightly improved influence on emergency hospital admissions for mental disorders^21^. A study in Italy had shown that short-term O_3_ exposure may increase the number of psychiatric hospital admissions eachday^18^.

There was evidence that inflammation and oxidative stress were key factors in the pathophysiology of diseases caused by air pollution, which was producedby increased production of pro-inflammatory mediators and reactive oxygen species as a result of exposure to various air pollutants^22^. Exposure to PM_2.5_ was associated with a reduction in the volume of the bilateral superior, middle, and medial frontal gyri cortex, as well aswhite matter in the frontal lobe with the largest clusters, temporal, parietal, and occipital lobes with small clusters^23^. These brain areas were involved in higher-level cognitive functions, such as working memory, episodic memory, and executive function. Long-term exposure to PM_2.5_ may hasten gray matter loss in elderly women, while the decreasedgray matter volume representedneutron atrophy and a decrease in the number of synaptic spines, dendritic branches, and synapses, which can severely impair cognitive performance^23^. The effects of PM on white matter volume were mainly concentrated in the frontal, parietal and temporal lobes, with regional distribution features. The decrease in white matter volume reflected oligodendrocytes and/or myelin degredation, which were related to cognitive function decline. Long-term exposure to PM_2.5_ is associated with lower total brain volume and more cryptogenic cerebral infarctions^24^. It was proved that PM_2.5_ can caused neuronal apoptosis and damage to the blood brain barrier^25^, that PM_2.5_ may be invovled in possible causative mechanisms of dementia. PM and ozone, two prevalent pollutants with different characteristics and reactivity, can stimulatedthe hypothalamic-pituitary-adrenal axis and trigger cortisol release, resulting in a neuroendocrine stress response^26^.

Our study had the following limitations. Firstly, this study was an ecological design study and could not avoid the ecological fallacy. Although time series studies had been used to control for some confounding factors, it was still not possible to completely rule out the influence of individual confounding factors, such as physical illness, occupation, lifestyle habits (smoking, drinking, liking outdoor activities, etc.). Estimating the average level of pollutants in the city to estimate the average exposure level of the population would result in certain errors, without considering the impact of indoor pollutant PM2.5. Secondly, due to the limited number of cases of some specific mental disorders, a more detailed grouping analysis was not carried out. Thirdly, the pathophysiology of mental disorders is not clear, but both hereditary and environmental factors have an impact on its pathogenesis. Our study did not investigate genetics-related factors. Finally, we have not done our best in controlling confounding factors, such as the patient's living environment, seasonal factors, light pollution, noise pollution, and other pollutant levels that may lead to individual differences in patients, which is also an important direction for our future inclusion and in-depth research.

In conclusion, short-term particulate pollution has a positive correlation with the daily hospital admission for mental disorders, especially among elderly men. At the same time, the combined effect of air pollutants and particulate pollutants might amplifythis effect.

## Supplementary Information

Supplementary Figure 1.

Supplementary Figure 2.

Supplementary Figure 3.

Supplementary Figure 4.

Supplementary Figure 5.

Supplementary Legends.

## Data Availability

The datasets generated and analyzed during the current study are available from the corresponding author on reasonable request.

## Abbreviations

ER: Excess risk
MD: Mental disorders
PM: Particulate matter
