# Supplementary figures and images for: Ecological correlation between short term exposure to particulate matter and hospitalization for mental disorders in Shijiazhuang, China
Source: Sci Rep. 2023 Jul 14;13:11412. doi: 10.1038/s41598-023-37279-7 (PMC10349047; doi:10.1038/s41598-023-37279-7)

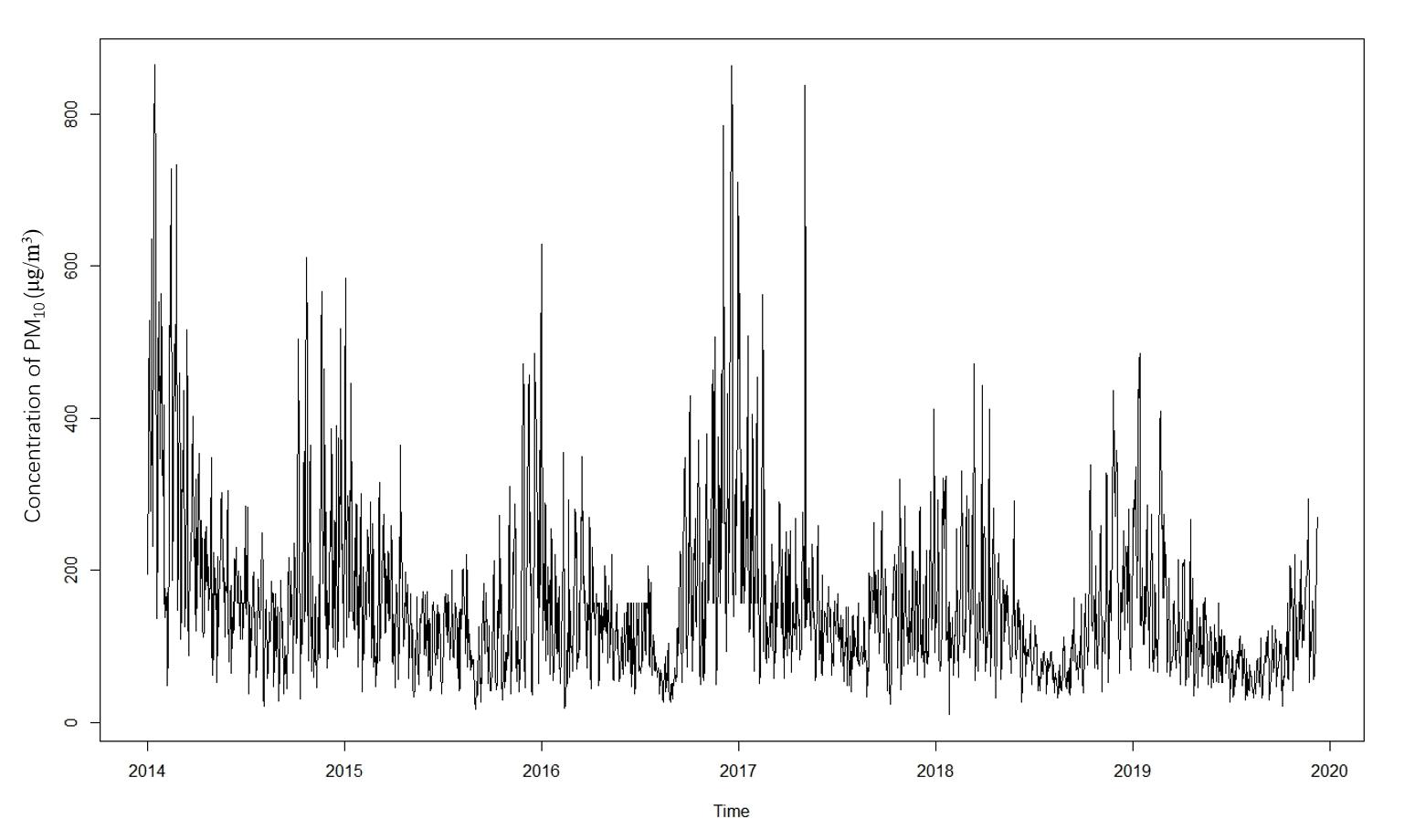

Supplement: Supplementary file 1 — Supplementary Figure 1. [file 41598_2023_37279_MOESM1_ESM.jpg]

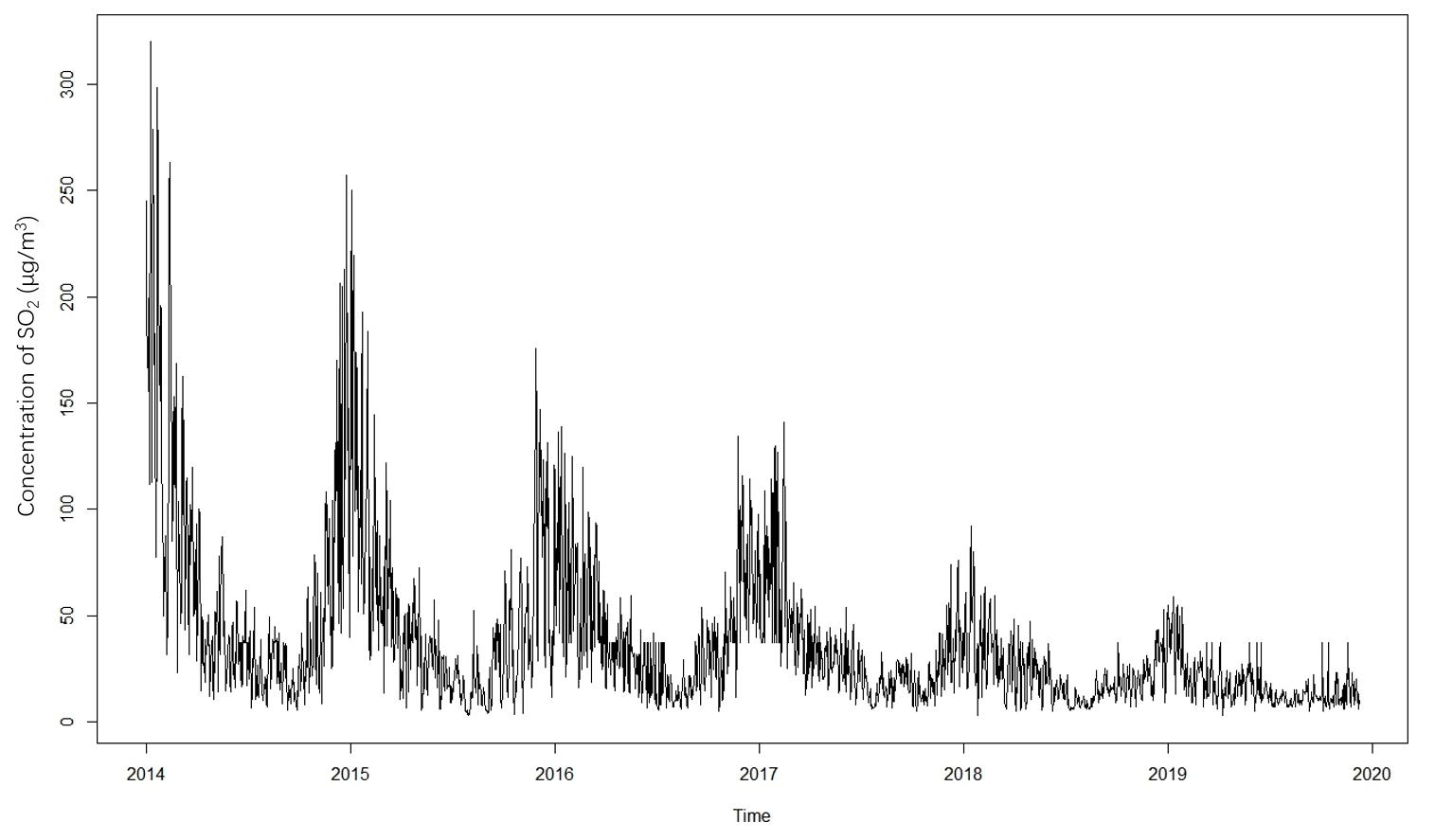

Supplement: Supplementary file 2 — Supplementary Figure 2. [file 41598_2023_37279_MOESM2_ESM.jpg]

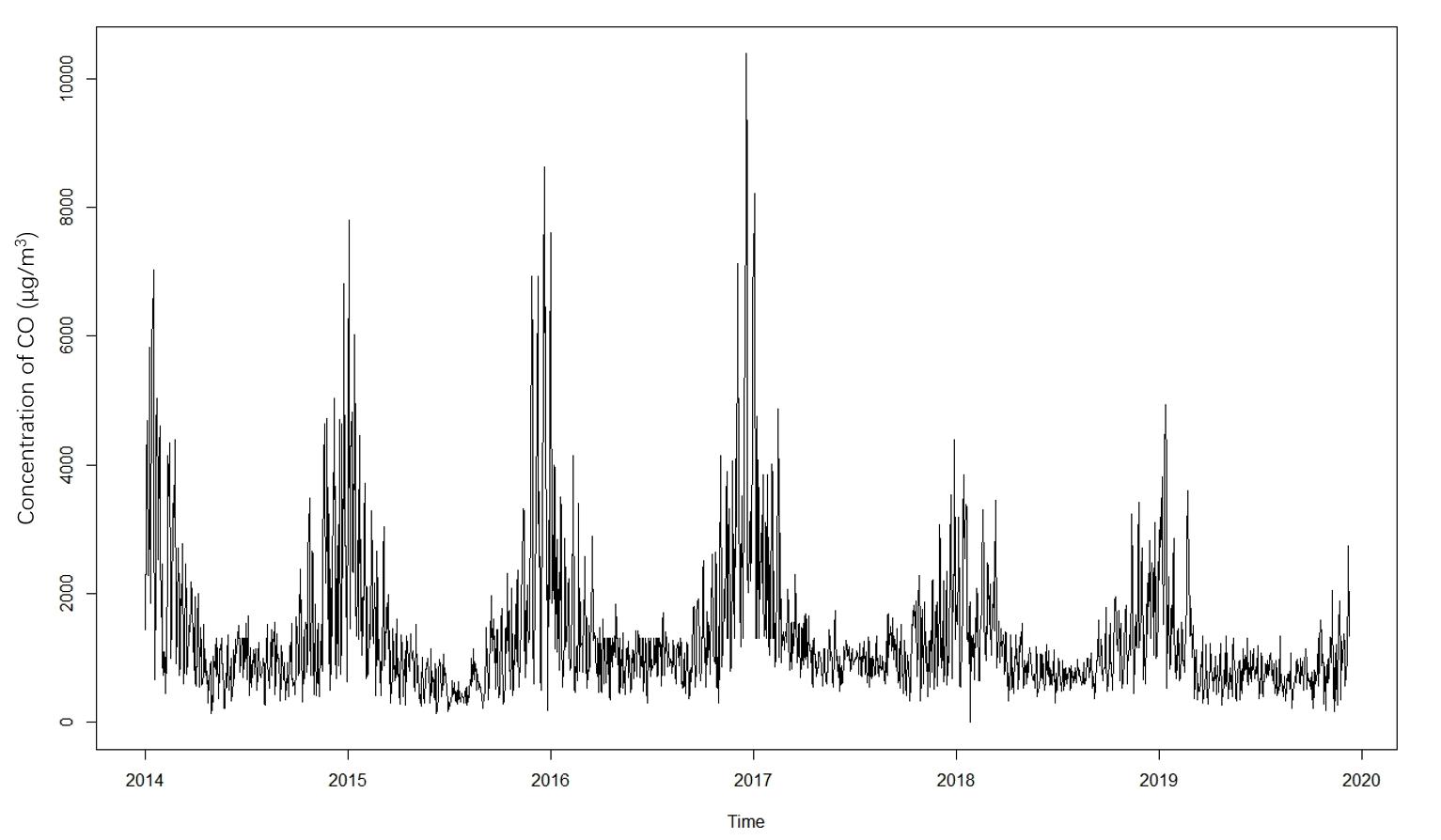

Supplement: Supplementary file 3 — Supplementary Figure 3. [file 41598_2023_37279_MOESM3_ESM.jpg]

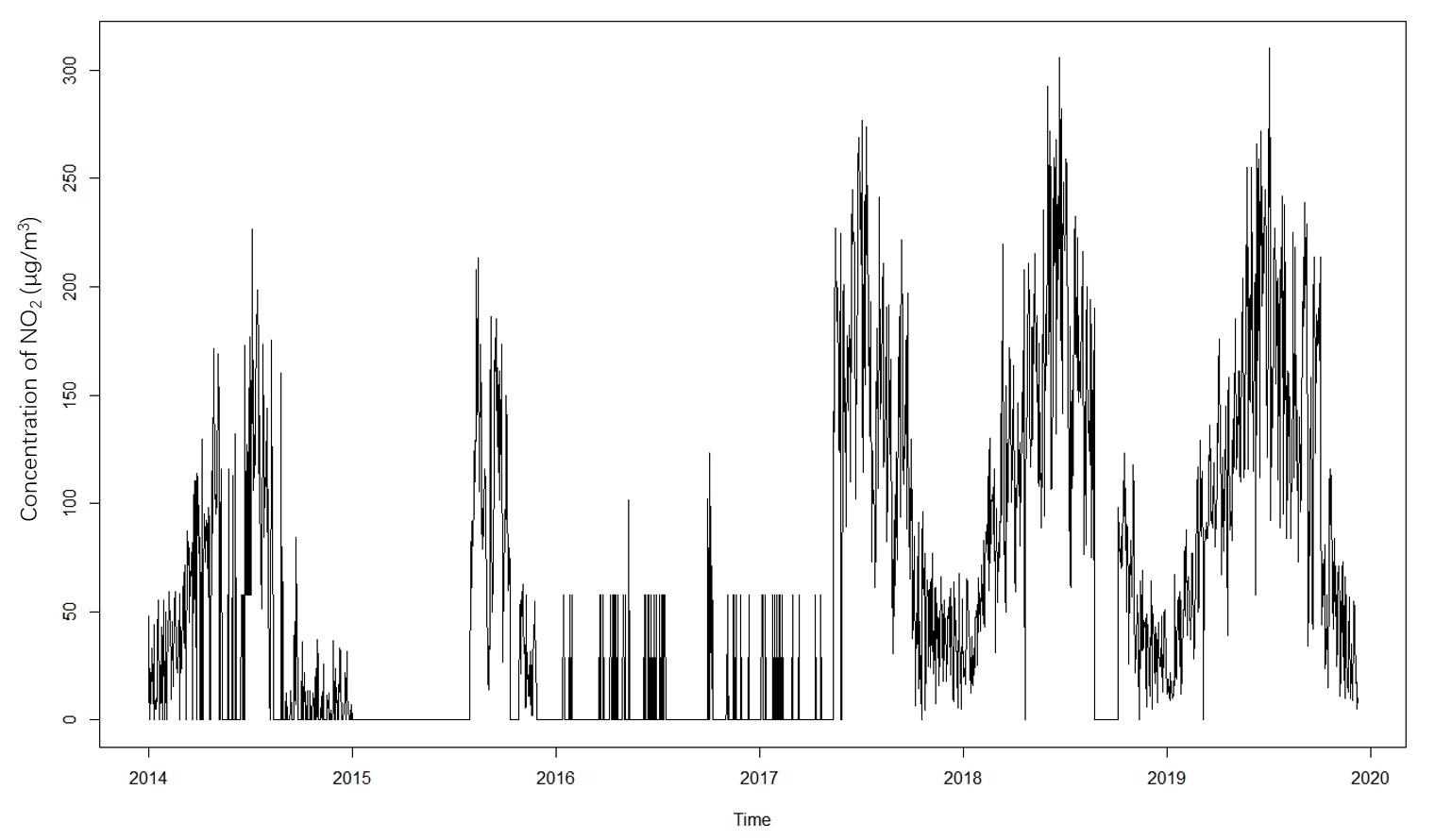

Supplement: Supplementary file 4 — Supplementary Figure 4. [file 41598_2023_37279_MOESM4_ESM.jpg]

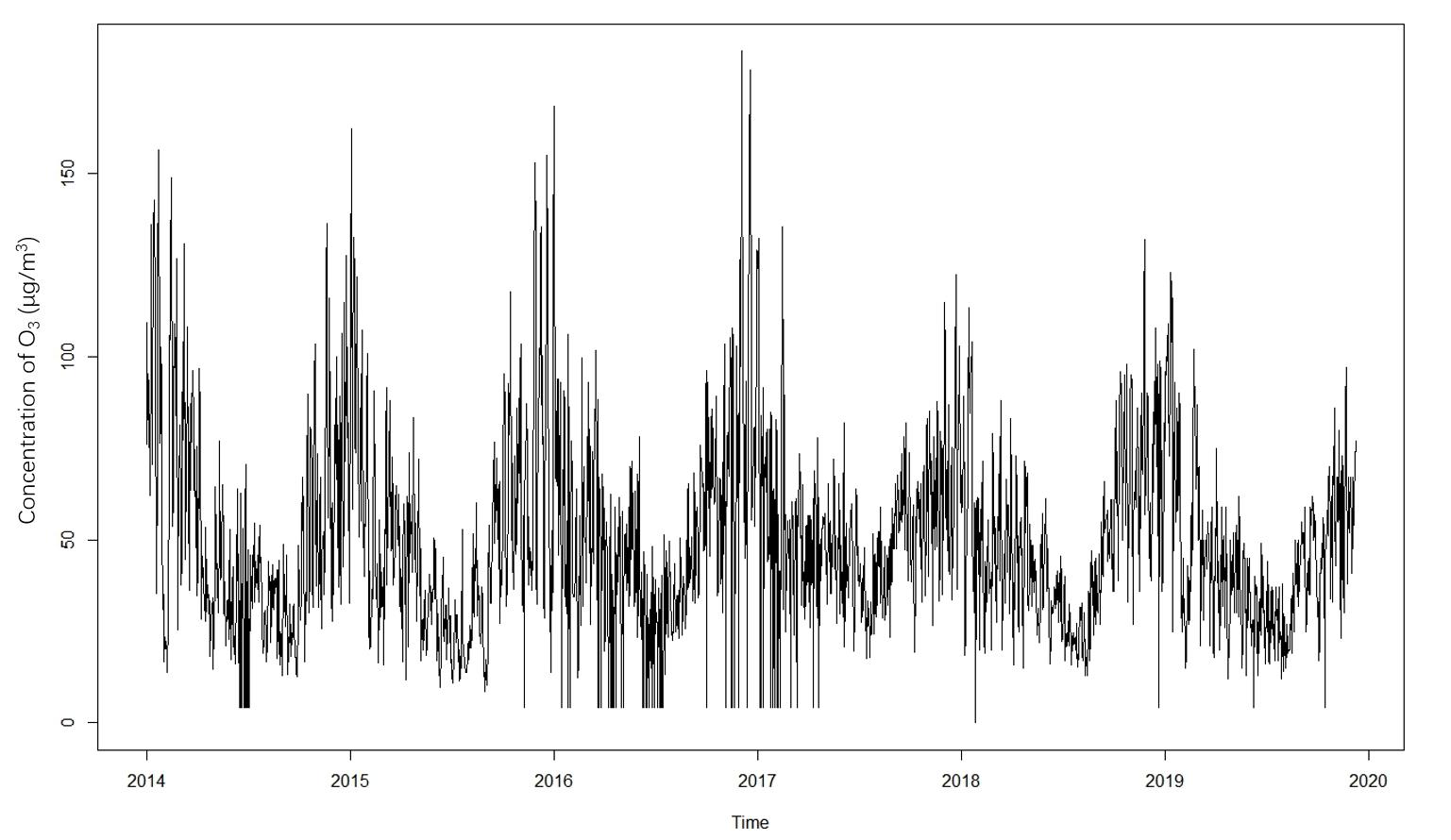

Supplement: Supplementary file 5 — Supplementary Figure 5. [file 41598_2023_37279_MOESM5_ESM.jpg]
