# Supplementary material for: Ecological correlation between short term exposure to particulate matter and hospitalization for mental disorders in Shijiazhuang, China
Source: Sci Rep. 2023 Jul 14;13:11412. doi: 10.1038/s41598-023-37279-7 (PMC10349047; doi:10.1038/s41598-023-37279-7)
Supplement: Supplementary file 6 — Supplementary Legends. [file 41598_2023_37279_MOESM6_ESM.docx]

**Supplementary figure legend**

Supplementary Figure 1. Distribution of PM_10_ from 2014 to 2019.

Supplementary Figure 2. Distribution of SO_2_ from 2014 to 2019.

Supplementary Figure 3. Distribution of CO from 2014 to 2019.

Supplementary Figure 4. Distribution of NO_2_ from 2014 to 2019.

Supplementary Figure 5. Distribution of O_3_ from 2014 to 2019.
